# Psychometric Properties of the Independent and Interdependent Self-Construal Questionnaire: Evidence From the Czech Republic

**DOI:** 10.3389/fpsyg.2021.564011

**Published:** 2021-06-03

**Authors:** David Lacko, Jiří Čeněk, Tomáš Urbánek

**Affiliations:** ^1^Department of Psychology, Faculty of Arts, Masaryk University, Brno, Czechia; ^2^Institute of Psychology, Czech Academy of Sciences, Brno, Czechia; ^3^Department of Social Studies, Faculty of Regional Development and International Studies, Mendel University in Brno, Brno, Czechia; ^4^Department of Information and Library Studies, Faculty of Arts, Masaryk University, Brno, Czechia

**Keywords:** individualism, collectivism, independent self-construal, interdependent self-construal, confirmatory factor analysis, psychometric properties, factor structure

## Abstract

This article introduces a validation study of the Czech version of an independent and interdependent self-construal questionnaire (SCS, Vignoles et al., 2016) conducted on 330 Czech subjects. In this study, the reliability, convergent validity and factor validity were verified. However, the confirmatory factor analysis revealed unsatisfactory factor structure (*RMSEA* = 0.053 [0.048, 0.057], *SRMR* = 0.080, *CFI* = 0.775, *TLI* = 0.755). These results are discussed with respect to other adaptations of individualism/collectivism scales in countries beyond typical West-East dichotomy. Hence, the article not only critically discusses the shortcoming of the Czech and original versions of the questionnaires, but also the general issues of the individualism-collectivism construct in the cross-cultural context as a whole.

## Introduction

Formulated in the 1970s by Hofstede, the cultural dimension of individualism/collectivism (I/C) has become a popular theoretical concept in cross-cultural psychology and a useful tool to structure and measure the psychological characteristics of members of various cultures (Bond, 2002). Consequently, I/C is used as a predictor for many other psychological and behavioral variables (Oyserman et al., 2002). The I/C dimension was originally defined at a national level as a single bipolar dimension. Hofstede (1983) defined individualism as the quality of a relationship between an individual and his or her immediate social environment (family, friends, community, etc.). The theory of independent (*de facto* individualism) and interdependent (*de facto* collectivism) self-construal (Markus and Kitayama, 1991) later became the dominant approach in individual I/C research. It is based on the “social orientation hypothesis” (Varnum et al., 2010), which states that cultures differ in social orientations and their development. While some (individualistic) cultures adopt an independent social orientation and tend to emphasize self-direction, self-expression and autonomy, other (collectivistic) cultures endorse the development of interdependent social orientation and emphasize harmony, relatedness and connection with others. Even though individualism is currently rising in most societies, the mentioned cross-cultural differences remain detectable (Santos et al., 2017).

Despite the popularity of the construct, there is unfortunately no widely accepted method of measuring the individual level of I/C. Oyserman et al. (2002) identified 27 I/C scales and performed a content analysis of I/C domains. They found seven individualism and eight collectivism components accounting for 88% of the items across the scales. None of these 27 scales can be considered a single standard of measurement. The overall agreement on I/C operationalization differs in the selected scales from component to component, which has drawn attention to the fragmentation of the concept of I/C and its operationalization.

The debate on the number of factors and their structure is still ongoing and the existing research has suggested that the concept of an independent and interdependent self might be one-dimensional (Hofstede, 1983), two-dimensional (Markus and Kitayama, 1991; Lu and Gilmour, 2007), three-dimensional (Kashima and Hardie, 2000; Noguchi, 2007), four-dimensional (Singelis et al., 1995), five-dimensional (Bartoš, 2010) or possibly even seven-dimensional (Vignoles et al., 2016).

Such ambiguity raises a question about the true underlying factor structure and therefore calls for further investigation on independent samples (Bollen, 1989b). The importance of this step is even more crucial in cross-cultural research, where securing the equivalence of constructs as well as the measurement invariance is necessary to be able to compare country or cultural group means (van de Vijver and Tanzer, 1997; van de Vijver and Leung, 2001; Čeněk and Urbánek, 2019). In order to acquire some evidence of structural equivalence, statistical methods such as Confirmatory Factor Analysis (CFA), Multi-Group Confirmatory Factor Analysis (MG-CFA), Measurement Invariance (MI), etc., need to be applied (Fischer and Karl, 2019). Performing CFA is vital in cross-cultural research as a first step in verifying the possibility of comparing results across different questionnaire translations. This article is focused mainly on this step. The next steps after setting the configural model should lie in constraining the factor structure, factor loadings and intercepts and thus verifying the configural, metric and scalar invariance measurement across various cultural groups.

## Individualism and Collectivism in the Czech Republic

Several previous studies have already been conducted on Czech participants with mixed results. Hofstede’s (Hofstede et al., 2010) approach assigned an index of 58 to the Czech Republic, suggesting a slightly above-average level of individualism. In cross-cultural comparisons, Czechs have been shown to be more individualistic than the rest of countries in Central Europe (Kolman et al., 2003). Dumetz and Gáboriková (2017) study confirmed that the Czech Republic is more individualistic than Slovakia, but others found the exact opposite result (Bašnáková et al., 2016). Furthermore, the Czech Republic seems to be less individualistic than the Netherlands (Bašnáková et al., 2016) and less collectivistic and, similarly, individualistic as East Asians (Lacko et al., 2020). Another study found that Czechs are not only more individualistic, but simultaneously also more collectivistic than Czech Vietnamese (Čeněk, 2015).

Unfortunately, these mixed results might be caused by the lack of valid and reliable tools to measure I/C, because adaptation attempts are relatively sparse for the Czech population. The first exception is an adaptation of INDCOL (Singelis et al., 1995) done by Bartoš (2010). However, his validation has a factor structure that does not fully correspond to the original scale, and its results are therefore not fully comparable in cross-cultural research. The psychometric properties of INDCOL are described in the next chapter.

The second exception, an attempt to adapt an I/C questionnaire into Czech, is a translation and cross-cultural verification of the Independent and Interdependent Self Scale (IISS; Lu and Gilmour, 2007) performed by Lacko and Čeněk (2020), who compared Czech and East Asian university students. Although the scale exhibited satisfactory reliability for both independent self-construal (Czech α = 0.815, Chinese α = 0.929) and interdependent self-construal (Czech α = 0.795, Chinese α = 0.906), the IISS showed configural non-invariance (*RMSEA* = 0.043 [0.025, 0.057], *SRMR* = 0.144, *CFI* = 0.636, *TLI* = 0.617). In their second study, performed on a larger sample consisting of only Czechs, they found similar unsatisfactory fit indices of IISS (*RMSEA* = 0.064 [0.061, 0.066], *SRMR* = 0.104, *CFI* = 0.460, *TLI* = 0.432).

The third exception is found in several adaptations of traditional methods measuring the cultural values, where individualism represents one of the cultural values. These Czech adaptations include VSM-94 (Value Survey Module 1994; Hofstede, 1994; adapted by Kolman et al., 2003), SVS (Schwartz Value Survey; Schwartz, 1992; adapted by Hnilica et al., 2006) and PVQ (Portraits Value Questionnaire; Schwartz et al., 2001; adapted in two studies by Řeháková, 2006; Anýžová, 2014). However, neither the original questionnaire manuals, nor the Czech adaptations of VSM-94 and SVS reported any relevant psychometric properties. The first study of PVQ reports only unsatisfactory internal reliability coefficients (α = 0.35–0.70; Řeháková, 2006). Even though the second study revealed acceptable MG-CFA fit indices (*CFI* = 0.924, *RMSEA* = 0.017), it suggested insufficient MI results across countries (metric MI Δ*CFI* = 0.009, scalar MI Δ*CFI* = 0.155) and these results were applied only to 10 out of 23 countries in total (Anýžová, 2014), which therefore raises doubts about the validity and reliability of those instruments.

As mentioned above, the two-dimensional model of the IISS failed in the factor structure validation on the Czech sample, and Hofstede’s one-dimensional model is claimed to be outdated and considered obsolete and invalid by many scholars (e.g., Singelis et al., 1995; McSweeney, 2002; Blodgett et al., 2008). Hence, the goal of this paper is to conduct an adaptation and psychometric analysis of a relatively new tool for individual level I/C measurement, the Self-Construal Scale (SCS; Vignoles et al., 2016; for a description see the Method section). Furthermore, we tried to verify its convergent validity with the current Czech adaptation of the Individualism-Collectivism Scale (INDCOL; Singelis et al., 1995; adapted by Bartoš, 2010).

## Materials and Methods

### Participants

Data were collected from 330 Czech participants. This number of participants should be satisfactory for several reasons: (1) our proposed models are simple and they are composed only of several first-order factors and indicators (Kline, 1998); (2) even though the rules of thumb are not fully reliable while planning research, they usually indicate an amount of 100–200 participants as an absolute minimum (Little, 2013; Brown, 2015) and moreover, as Kline (1998) pointed out, the number of about 200 participants is not only very often used in the SEM framework, but it might also be reliable in certain circumstances; (3) no missing values were observed in our dataset (Brown, 2015; Kline, 1998); (4) I/C scales usually yield high internal-consistency reliability which decreases demands on sample size (Brown, 2015; Kline, 1998); (5) in single group models standard errors are significantly reduced with more than 150 responses (Little, 2013); and (6) our models yielded a huge number of degrees of freedom (Hoyle, 2012; Kline, 1998).

The research sample was 77% (*n* = 254) female. The participants were 18–65 years old (*M* = 24.29, *SD* = 6.536). Regarding the field of study, or vocation, 34.5% (*n* = 114) of the participants were psychologists/students of psychology, followed by students of/employees in the field of languages and history and of international studies (both 8.8%). Concerning the level of education, 58.8% (*n* = 194) had completed high school and 39.7% (*n* = 131) had achieved a university degree. Regarding the participants’ religion, political preference and family status, most of them identified themselves as an atheist (43.6%), had no political preference (40.6%; or were liberals 31.2%) and were single (58.5%). The comprehensive descriptive statistics are shown in Table 1:

**TABLE 1:** Demographic characteristic of sample.

Variable	Choice	Frequency
Gender	Man	76 (23.03%)
	Woman	254 (76.97%)
Age	Range	18–65
	Mean (SD)/median (IQR)	24.293 (6.536)/23 (4)
Family status	Single	193 (58.485%)
	In partnership	105 (31.818%)
	Married	24 (7.273%)
	Divorced	7 (2.121%)
	Widow	1 (0.303%)
Education	Primary school	2 (0.606%)
	High school	194 (58.788%)
	Higher vocational school	3 (0.909%)
	University	131 (39.697%)
Field of study/occupation	Psychology	114 (34.545%)
	International studies	29 (8.788%)
	IT	12 (3.636%)
	Pedagogy	19 (5.758%)
	Regional development	16 (4.848%)
	Information studies and librarianship	9 (2.727%)
	Languages and history	29 (8.788%)
	Other	102 (30.909%)
Salary of family during childhood	1300 CZK and less	21 (6.364%)
	1300–6500 CZK	84 (25.455%)
	6500–13000 CZK	130 (39.394%)
	13000–33000 CZK	79 (23.939%)
	More than 33000 CZK	16 (4.848%)
Religion	Atheist	144 (43.636%)
	Christianity	79 (23.939%)
	Spiritually based person	92 (27.879%)
	Other	15 (4.545%)
Political opinions	No preference	134 (40.606%)
	Liberalism	103 (31.212%)
	Environmentalism and green politics	45 (13.636%)
	Conservatism	24 (7.273%)
	Socialism	10 (3.030%)
	Nationalism	5 (1.515%)
	Anarchy	4 (1.212%)
	Other	5 (1.515%)
Number of siblings	0	51 (15.455%)
	1	161 (48.788%)
	2	78 (23.636%)
	3	26 (7.879%)
	4 and more	14 (4.242%)

### Procedure

Data collection was conducted between December 2018 - February 2019. The participants were mostly gathered through university social groups and social websites (i.e., the non-probability convenience sampling method) which resulted in a research sample with an over-represented student part of the population compared to other groups. The participants were informed about the ethical aspects of the research, especially data anonymization, their voluntary participation and the option to end the questionnaire at any time without giving a reason. In order to proceed further with the administration, they had to consent with their participation in the research. All items were administered randomly to avoid possible response biases caused by context influences and preceding questions (for review, see Uskul and Oyserman, 2006). The whole testing procedure took approximately 20 min.

In order to minimize any potential method bias caused by an imperfect translation procedure (van de Vijver and Hambleton, 1996) a parallel translation method was applied. The English original Self-Construal Scale (SCS) was independently translated by two bilinguals with backgrounds in social sciences. Both translations were subsequently compared and, in the case of any inconsistencies, discussed by the authors of the study until an agreement on the formulation was reached. We put a special emphasis on minimizing any potential shifts of meaning between the English and Czech versions of the scale. The Individualism-Collectivism Scale (INDCOL) was used in the original Czech version (Bartoš, 2010). Both scales were administered online. In addition to SCS and INDCOL, all relevant socio-demographic variables were collected (see Table 1).

### Measures

#### The Self-Construal Scale (SCS)

The SCS was developed by Vignoles et al. (2016) and validated on 9573 (Study 1, n = 2294; Study 2, n = 7279) participants across 55 cultural groups in 33 nations. The SCS is primarily based on the concept of independent and interdependent self (Markus and Kitayama, 1991). The authors built on other traditional I/C scales during the formulation of its items (e.g., Singelis et al., 1995).

The SCS consists of thirty-eight, nine-point, Likert-type numerical items scaled from 1 (not at all) to 9 (exactly), with three intermediate anchor-points (3 – a little, 5 – moderately, 7 – very well). The SCS contains half reversed items, which should enhance the validity of the factor structure and minimize the acquiescence bias (Smith et al., 2013).

The authors used exploratory and confirmatory factor analytic techniques, MG-CFA, multilevel analysis and other statistical procedures (such as modeling acquiescence as a common method factor in CFA or ipsatization for reliability estimation) in their validation study. Even though the authors did not perform an analysis of MI, they discussed it in relation to the items’ factor loadings, which in their opinion suggested a satisfactory invariance. However, no reliability estimation was performed in the validation study. The SCS was also tested for response biases in their follow-up study (cf. Smith et al., 2016) as well as concurrent validity with the I/C dimensions measured by individualism values and in-group collectivism practices, where the *r* coefficients were between 0.425 and 0.752.

Using Principal Components Analysis (PCA) and Principal Axis Factoring (PAF) in the first study, the authors identified seven dimensions of the SCS, namely “Self-reliance vs. Dependence on others,” “Self-containment vs. Connection to others,” “Difference vs. Similarity,” “Commitment to others vs. Self-interest,” “Consistency vs. Variability,” “Self-direction vs. Receptiveness to influence” and “Self-expression vs. Harmony.” The CFA partially confirmed the factor structure in the second study. The authors presented two respective models: model 1, which was comprised of 38 items, and model 2, with 26 items. The first model yielded good fit indices despite an insufficient *CFI* (*SRMR* = 0.050; *RMSEA* = 0.046; *CFI* = 0.790). However, the authors claimed that a 0.90 threshold for CFI is often unreachable and unrealistic in multidimensional questionnaires used in various cultural samples and they justified a *CFI* value near 0.80 as acceptable for cross-cultural multidimensional questionnaires. The second model showed better fit indices (*SRMR* = 0.033, *RMSEA* = 0.033, *CFI* = 0.922) and can be considered valid from the point of view of factor structure. Nevertheless, this model only has 26 items and one subscale is consequently comprised of only two items, whereas an unabbreviated model contains four to six items per subscale. Our view is that 26 items are insufficient for a seven-dimensional questionnaire, and we therefore focused on the longer version of the SCS. At the same time, we concur with the authors that the SCS is currently one of the most comprehensive tests available for I/C dimension measurement.

#### The Individualism-Collectivism Scale (INDCOL)

The INDCOL was introduced by Singelis et al. (1995) and later improved by Triandis and Gelfand (1998). The original scale contains 32 items, and the improved and shortened version contains 27 items. All items are nine-point, Likert-type questions. Both questionnaires measure four dimensions: horizontal collectivism (HC – empathy, cooperation, sociability), horizontal individualism (HI – independence, uniqueness, self-sufficiency), vertical collectivism (VC – submissiveness) and vertical individualism (VI – competitiveness). The validation study was conducted on 267 participants by Singelis et al. (1995). The reliability of scales was not ideal (HI α = 0.67, VI α = 0.74, HC α = 0.74, VC α = 0.68) nor were the CFA fit indices [χ2 (458) = 898.88, *GFI* = 0.79, *AGFI* = 0.75, *RMSR* = 0.089]. Based on the CFA results, the item pool was reduced from 94 to 32 items. The questionnaire was improved by Triandis and Gelfand (1998) on 543 participants in total (Study 1, n = 326; Study 2, n = 127; Study 3, n = 90). They selected 27 items with the highest factor loadings and also reported higher reliability coefficients (HI α = 0.81, VI α = 0.82, HC α = 0.80, VC α = 0.73). The 27 INDCOL items also showed good convergent and divergent validity through correlations with I/C scenarios. However, they did not repeat the CFA for the 27-item questionnaire, nor did they perform MI.

The Czech validation study was conducted by Bartoš (2010) on 1081 participants. He modified the nine-point, Likert type items to seven-points and reduced the number of items to 24. He applied Exploratory Factor Analysis (EFA) to examine the factor structure of the Czech version of the INDCOL and found five factors. He separated HI into HI1 (uniqueness; 3 items) and HI2 (independence; 2 items). VI (7 items), HC (7 items) and VC (5 items) remained the same as in the original study. He also conducted reliability estimates, but two scales did not meet the minimum criteria (VI α = 0.79, HI1 α = 0.71, HI2 α = 0.60, VC α = 0.63, HC α = 0.76). Although the Czech version of the scale seems to have limited psychometric properties, we decided to use it in this study for two reasons: (1) to verify its factor structure as reported by Bartoš (2010) using CFA on an independent Czech sample, and (2) to test its convergent validity with SCS, because it is, despite its limitations, the only available criteria for Czech samples.

### Analytical Procedure

In order to examine the factor structure of both questionnaires, we performed a CFA with a robust, weighted, least square mean and variance (WLSMV) estimator, which is suitable for ordinal and non-Gaussian distributed data from Likert-type scales (Finney and DiStefano, 2013), because according to multivariate Henze-Zirkler tests, data were non-normally distributed at the subscale level (univariate Shapiro-Wilk tests confirmed these findings at the item level) for both questionnaires, and which is also less biased than robust maximum likelihood (MLR; Li, 2016). As the criteria for evaluating a good model fit, many more or less strict cut-offs are used. We used the Tucker-Lewis Index (*TLI*) ≥ 0.95, Comparative Fit Index (*CFI*) ≥ 0.95, Root Mean Square Error of Approximation (*RMSEA*) ≤ 0.60, Standardized Root Mean Square Residual (*SRMR*) ≤ 0.80 (Hu and Bentler, 1999) and Adjusted Goodness-of-Fit Index (*AGFI*) ≥ 0.90 (Hooper et al., 2008) fit indices for the evaluation of a good model fit in this study.

Internal consistency of subscales was assessed with Cronbach’s α and McDonald’s ω. We used the 0.70 threshold of internal consistency as a satisfactory indicator of reliability. We also performed a reliability analysis with ipsatization in order to reduce culture-specific response and acquiescence biases (Fischer, 2004; Fischer and Milfont, 2010). Standardized within-subject ipsative scores were calculated for each item of each individual according to the following formula:

$$i\,p\,s\,a\,t\,i\,v\,e\,s\,c\,o\,r\,e=\frac{r\,e\,s\,p\,o\,n\,s\,e-M\,o\,f\,s\,c\,a\,l\,e\,f\,o\,r\,e\,a\,c\,h\,i\,n\,d\,i\,v\,i\,d\,u\,a\,l}{S\,D\,o\,f\,s\,c\,a\,l\,e\,o\,r\,e\,a\,c\,h\,i\,n\,d\,i\,v\,i\,d\,u\,a\,l}.$$

Convergent validity between and within measures was verified with nonparametric Spearman’s correlation analyses, while each subscale score was entered into analysis as arithmetic mean. We interpreted correlation coefficients higher than 0.50 as indicators of minimally acceptable convergent validity and coefficients higher than 0.70 as sufficient evidence for convergent validity (Carlson and Herdman, 2010). The statistical analysis was conducted in *R* (v 3.6.1; R Core Team, 2020), specifically the packages *lavaan* (Rosseel, 2012), *semTools* (Jorgensen et al., 2018), *psych* (Revelle, 2020), *ShinyItemAnalysis* (Martinkova and Drabinova, 2018), and *MVN* (Korkmaz et al., 2014).

## Results

The descriptive statistics (means, standard deviations, skewness and kurtosis) of all scales are shown in Table 2. The item analysis within classical test theory approach (i.e., descriptive statistics of items, several types of discrimination, etc.) is reported in Supplementary Appendix II.

**TABLE 2:** The descriptive statistics of subscale scores.

Scale	Subscale	*M* [95% *CI*]	*SD*	Skewness	Kurtosis
SCS	Difference vs. Similarity	5.59 [5.43, 5.75]	1.45	−0.143	−0.423
	Self-containment vs. Connection to others	4.28 [4.14, 4.43]	1.37	0.501	−0.122
	Self-direction vs. Receptiveness to influence	6.03 [5.88, 6.18]	1.40	−0.036	−0.726
	Self-reliance vs. Dependence on others	6.61 [6.44, 6.77]	1.50	−0.658	0.521
	Consistency vs. Variability	5.09 [4.91, 5.27]	1.67	−0.044	−0.460
	Self-expression vs. Harmony	5.02 [4.89, 5.16]	1.28	−0.121	−0.179
	Self-interest vs. Commitment to others	4.66 [4.51, 4.81]	1.36	0.412	−0.167
INDCOL	Vertical individualism	3.81 [3.68, 3.93]	1.15	0.240	−0.273
	Horizontal collectivism	5.21 [5.12, 5.31]	0.86	−0.498	0.247
	Vertical collectivism	3.41 [3.30, 3.52]	1.01	0.136	−0.244
	Horizontal individualism 1	4.88 [4.75, 5.01]	1.21	−0.623	−0.114
	Horizontal individualism 2	4.21 [4.07, 4.36]	1.34	0.107	−0.556

**M*, mean; *SD*, standard deviation; *CI*, confidence intervals.*

### Factor Structure

The Czech version of the SCS showed satisfactory *RMSEA* and *SRMR*. The relative chi-square (χ^2^/df) was 1.913, which suggested a good global fit of the model (Kline, 1998). However, the model showed unsatisfactory *CFI* and *TLI* values. Nevertheless, we would like to emphasized that *CFI* of the Czech version of the SCS was almost the same as the *CFI* of the Vignoles et al. (2016) original version (see Table 3). A common factor with acquiescence as a common method factor was used on the reversed items following the procedure used by Vignoles et al. (2016) in order to reduce acquiescence bias (see Welkenhuysen-Gybels et al., 2003). This model also did not yield satisfactory fit indices.

**TABLE 3:** The SCS and SCS modified model fit indices compared to the original version by Vignoles et al. (2016).

Model	Chi-Square	*p*	*RMSEA* [90% *CI*]	*SRMR*	*CFI*	*TLI*	*AGFI*
CZ SCS 1	χ^2^ (644) = 1232.107	<0.001	0.053 [0.048, 0.057]	0.080	0.775	0.755	0.920
CZ SCS 2	χ^2^ (625) = 1125.036	<0.001	0.049 [0.045, 0.054]	0.073	0.809	0.785	0.931
Original SCS	NR	NR	0.046 [NR]	0.050	0.790	NR	NR
CZ SCS Mod.	χ^2^ (632) = 1011.010	<0.001	0.043 [0.038, 0.048]	0.066	0.855	0.839	0.943
CZ SCS (1 second-order factor)	χ^2^ (658) = 1392.214	<0.001	0.058 [0.054, 0.062]	0.093	0.720	0.700	0.893
CZ SCS (bifactor)	χ^2^ (606) = 975.433	<0.001	0.043 [0.038, 0.048]	0.060	0.859	0.836	0.951
CZ SCS (2 factors)	χ^2^ (664) = 2013.118	<0.001	0.079 [0.075, 0.083]	0.121	0.485	0.454	0.824
CZ SCS 3	χ^2^ (605) = 975.774	<0.001	0.043 [0.038, 0.048]	0.059	0.858	0.835	0.953

*NR, not reported; CZ SCS 1, Czech version of SCS; CZ SCS 2, Czech version of SCS with one common factor with acquiescence as a common method factor on reversed items; Original SCS, original version of SCS; CZ SCS Mod., Czech version of SCS with allowed cross-loadings; CZ SCS (1 second-order factor), Czech version of SCS with one higher-order factor; CZ SCS (bifactor), Czech version of SCS with one general factor; CZ SCS (2 factors), Czech version of SCS two-dimensional model; CZ SCS 3, Czech version of SCS with two common factors with acquiescence as a common method factor separately on reversed and positive items; *p, p-*value; χ^2^, chi-square; *RMSEA*, root mean square error of approximation; *SRMR*, standardized root mean square residual; *CFI*, Comparative Fit Index; *TLI*, Tucker-Lewis Index; *CI*, confidence intervals; AGFI, adjusted goodness of fit index.*

Almost all of the items’ factor loadings besides three instances were above the recommended 0.40 threshold (Fornell and Larcker, 1981). Even if we take into consideration the stricter thresholds, for instance 0.50 (Hair et al., 2018), we would obtain only four more such instances. Furthermore, the current factor loadings often being higher than the originals obtained by Vignoles et al. (2016; see Supplementary Appendix I). All item parameters, covariances (with two exceptions) and variances were statistically significant). Therefore, no items had to be removed from the model in order to improve its fit.

An analysis of the potential cross-loadings with a modification index (*mi*) and expected parameter change (*epc*) could bring deeper insight into model misfit. We found that item 32 (for items wording see Supplementary Appendix I) from “Self-expression vs. Harmony” had potential cross-loadings on subscales “Self-containment vs. Connection to others” (*mi* = 155.075, *epc* = 0.869), “Self-interest vs. Commitment to others” (*mi* = 148.989, *epc* = 1.048) and “Consistency vs. Variability” (*mi* = 87.303, *epc* = −0.627). Analogously, item 15 from Self-direction vs. Receptiveness to influence had potential cross-loadings on subscales “Self-containment vs. Connection to others” (*mi* = 73.090, *epc* = 1.079), “Consistency vs. Variability” (*mi* = 68.539, *epc* = −0.746) and “Self-interest vs. Commitment to others” (*mi* = 57.856, *epc* = 1.239). Item 35 from “Self-interest vs. Commitment to others” had potential cross-loadings on “Self-direction vs. Receptiveness to influence” (*mi* = 61.102, *epc* = 1.124), “Self-expression vs. Harmony” (*mi* = 43.283, *epc* = 0.789) and “Self-containment vs. Connection to others” (*mi* = 39.786, *epc* = −1.486). Finally, item 25 from “Consistency vs. Variability” had potential cross-loadings on subscales “Self-direction vs. Receptiveness to influence” (*mi* = 55.498, *epc* = 0.563), “Self-interest vs. Commitment to others” (*mi* = 49.360, *epc* = 0.453) and “Self-containment vs. Connection to others” (*mi* = 48.192, *epc* = 0.485). These findings are discussed in detail in the Discussion section.

The analysis of modification indices showed that the SCS contained multiple cross-loaded items. If all of the above-mentioned cross-loadings are included in the model (CZ SCS Mod.; see Table 2 above), the majority of fit indices would be better than the original study by Vignoles et al. (2016). However, even these improved fit indices could still be considered unsatisfactory. Furthermore, using an exploratory approach (i.e., not confirmatory, cf. Bollen, 1989a; Byrne, 2010) we proposed four alternative models with individualism dimension as a one second-order factor of all subscales (i.e., CZ SCS 1 second-order factor), with individualism as a general factor in the bifactor model (i.e., CZ SCS bifactor), with individualism (non-reversed items) and collectivism subscales (reversed items in two-dimensional model (i.e., CZ SCS 2 factors) and with two common factors with acquiescence as a common method factor separately on reversed and positive items (i.e., CZ SCS 3, see Table 2). None of these models fit the data well.

The same CFA procedure was applied for the INDCOL scale with similar results as the SCS. The relative chi-square (χ^2^/df) was 2.118, which suggested a good global fit. The *RMSEA* and *SRMR* were satisfactory, however, the *CFI* and *TLI* were not. Hence, the current CFA results did support neither the 5-factor configural model provided by Bartoš (2010) nor the original 4-factor model provided by Singelis et al. (1995; see Table 4).

**TABLE 4:** The INDCOL fit indices compared to the original version by Singelis et al. (1995).

Model	Chi-Square	*p*	*RMSEA* [90% CI]	*SRMR*	*CFI*	*TLI*	*AGFI*
CZ INDOCL (5 factors)	χ^2^(242) = 521.126	<0.001	0.059 [0.052, 0.066]	0.074	0.779	0.748	0.932
CZ INDOCL (4 factors)	χ^2^(246) = 537.226	<0.001	0.060 [0.053, 0.067]	0.077	0.769	0.741	0.929
Original INDCOL	χ^2^(458) = 898.88	NR	0.089 [NR]	NR	NR	NR	0.75

*NR, not reported; CZ INDCOL, Czech version of INDCOL; Original INDCOL, original version of INDCOL; *p*, *p*-value; χ^2^, chi-square; RMSEA, root mean square error of approximation; SRMR, standardized root mean square residual; CFI, Comparative Fit Index; TLI, Tucker-Lewis Index; CI, confidence intervals; ECVI, expected cross-validation index; AGFI, adjusted goodness of fit index.*

### Reliability

Concerning the reliability of the SCS, Cronbach’s α varied between 0.667 and 0.855, while McDonald’s ω fell between 0.651 and 0.854 (see Table 5). The values of both coefficients showed satisfactory internal consistency in most of the subscales. Three subscales were slightly below the minimum threshold of 0.70. In case of ipsatization, α varied between 0.265 and 0.786. The results with ipsative scores were less satisfactory than the raw score results. This suggested that the questionnaire’s items might have been potentially influenced by a response bias (especially the subscales “Self-direction vs. Receptiveness to influence” and “Self-expression vs. Harmony”).

**TABLE 5:** Reliability estimations of the Czech version of the SCS subscales.

Dimension	*α SCS* (ipsatized)	*ω SCS*
Difference vs. Similarity	0.759 (0.571)	0.771
Self-containment vs. Connection to others	0.697 (0.634)	0.707
Self-direction vs. Receptiveness to influence	0.670 (0.265)	0.674
Self-reliance vs. Dependence on others	0.772 (0.624)	0.774
Consistency vs. Variability	0.855 (0.782)	0.854
Self-expression vs. Harmony	0.651 (0.291)	0.651
Self-interest vs. Commitment to others	0.763 (0.463)	0.764

*α, Cronbach’s alpha; ω, McDonald’s omega.*

Concerning the reliability of the INDCOL, α varied between 0.502 and 0.812 (for ipsative α between 0.432 and 0.642), while ω fell between 0.483 and 0.814 (see Table 6). The values of both coefficients demonstrated satisfactory internal consistency only for the VI and HC subscales. VC and HI demonstrated unsatisfactory internal consistency, and the ipsative scores suggested that they might have been influenced by a response bias.

**TABLE 6:** Reliability estimations of the Czech version of the INDCOL subscales.

Dimension	α INDCOL (ipsatized)	ω INDCOL
Vertical individualism (VI)	0.812 (0.642)	0.814
Horizontal individualism 1 (HI1)	0.564 (0.432)	0.483
Horizontal individualism 2 (HI2)	0.502 (0.510)	0.510
Vertical collectivism (VC)	0.589 (0.435)	0.577
Horizontal collectivism (HC)	0.739 (0.632)	0.737

*α, Cronbach’s alpha; ω, McDonald’s omega.*

### Convergent Validity Between Measures

In the following section, the results of the correlation analyses between subscales of the original and adapted version of SCS, between subscales of the original and adapted version of INDCOL, and between scales of SCS and INDOL are reported. A comparison of Spearman’s ρ to the original correlation coefficients (by Vignoles et al., 2016) in the subscales is shown in Table 7. All differences between obtained and original coefficients were smaller than 0.250. Besides a few exceptions, the Czech version showed relatively similar patterns of correlations to the original version. All of these associations were statistically significant and ranged from small to medium effect sizes (with exceptions of two insignificant associations and one association with high effect size).

**TABLE 7:** Comparison of the estimated correlations in the Czech version (below diagonal) and the original version (above diagonal) of the SCS by Vignoles et al. (2016).

Dimension	1	2	3	4	5	6	7
1. Difference vs. Similarity	−	0.112	0.288	0.436	0.136	0.401	0.214
2. Self-containment vs. Connection to others	0.143** [0.035, 0.247]	−	0.625	−0.075	−0.219	0.330	0.557
3. Self-direction vs. Receptiveness to influence	0.299*** [0.197, 0.394]	0.405*** [0.311, 0.492]	−	0.328	−0.002	0.417	0.435
4. Self-reliance vs. Dependence on others	0.240*** [0.135, 339]	0.125* [0.017, 0.229]	0.479*** [0.391, 0.558]	−	0.301	0.132	0.104
5. Consistency vs. Variability	0.267*** [0.164, 0.364]	−0.008 [−0.116, 0.100]	0.243*** [0.139, 0.342]	0.193*** [0.086, 0.294]	−	0.252	−0.141
6. Self-expression vs. Harmony	0.450*** [0.360, 0.532]	0.246*** [0.142, 0.345]	0.424*** [0.331, 0.509]	0.180** [0.074, 0.283]	0.299*** [0.198, 0.394]	−	0.366
7. Self-interest vs. Commitment to others	0.334*** [0.234, 0.426]	0.619*** [0.547, 0.681]	0.468*** [0.379, 0.548]	0.300*** [0.199, 0.395]	0.043 [−0.065, 0.150]	0.443*** [0.352, 0.526]	−

*p < 0.05, **p < 0.01, ***p < 0.001.

A comparison of the Spearman’s ρ correlations and original correlations of the Czech INDCOL version among the subscales is shown in Table 8. Even though current correlations were generally higher than correlations reported by Bartoš (2010), the corelations coefficients were still rather small. We also observed three differences between original coefficients and coefficients obtained in this study which were higher than 0.250. However, our results appear to be more in line with the I/C theory, because negative correlations between the HC (collectivism) and individualistic subscales (VI and HI2) were observed (instead of positive as reported by Bartoš, 2010).

**TABLE 8:** Comparison of the estimated correlations in the current (below diagonal) and original study (above diagonal) of the Czech version of INDCOL by Bartoš (2010).

Dimension	VI	HI1	HI2	VC	HC
VI	−	NR	NR	NR	0.19***
HI1	0.292*** [0.190, 0.388]	−	NR	−0.13***	−0.12***
HI2	0.241*** [0.136, 0.340]	0.112* [0.004, 0.217]	−	−0.13***	0.11***
VC	−0.083 [−0.189, 0.025]	−0.214*** [−0.314, −0.108]	−0.214*** [−0.315, −0.109]	−	NR
HC	−0.217*** [−0.318, −0.112]	−0.157** [−0.261, −0.050]	−0.308*** [−0.403, −0.207]	0.402*** [0.308, 0.489]	−

*p < 0.05, **p < 0.01, ***p < 0.001; NR, not reported.

Relationships were also expected between the SCS subscales and the INDCOL subscales as a demonstration of convergent validity. We assumed that HC and VC (i.e., collectivism) should be negatively correlated with all SCS subscales, whereas HI1, HI2 and VI (i.e., individualism) should correlate positively. As shown in Table 9, our expectations about directions were confirmed. However, these *r*_*s*_ coefficients were relatively small, and some of them non-significant. Only four associations were higher than 0.50 threshold (“difference vs. similarity” and “horizontal individualism: uniqueness”; “self-direction vs. receptiveness to influence” and “vertical collectivism”; “self-containment vs. connection to others” and “horizontal collectivism”; and “self-interest vs. commitment to others” and “horizontal collectivism”); none of them were above the recommended 0.70 value. Hence, these results do not support the assumption of the convergent validity of the SCS and INDCOL. It appears that both scales measure slightly different constructs.

**TABLE 9:** Coefficients of Spearman’s rho in the SCS and INDCOL.

Dimension	VI	HI1	HI2	VC	HC
Difference vs. Similarity	0.269*** [0.166, 0.366]	0.586*** [0.510, 0.652]	0.053 [−0.055, 0.160]	−0.347*** [−0.439, −0.249]	−0.098 [−0.204, 0.010]
Self-containment vs. Connection to others	0.154** [0.047, 0.258]	0.160*** [0.053, 0.263]	0.273*** [0.170, 0.370]	−0.389*** [−0.477, −0.293]	−0.561*** [−0.631, −0.482]
Self-direction vs. Receptiveness to influence	0.086 [−0.023, 0.192]	0.234*** [0.130, 0.334]	0.290*** [0.186, 0.386]	−0.552*** [−0.623, −0.473]	−0.285*** [−0.381, −0.182]
Self-reliance vs. Dependence on others	0.210*** [0.104, 0.311]	0.277*** [0.174, 0.374]	0.263*** [0.159, 0.361]	−0.324*** [−0.417, −0.223]	−0.155*** [−0.258, −0.047]
Consistency vs. Variability	−0.061 [−0.168, 0.047]	0.090 [−0.018, 0.196]	0.062 [−0.046, 0.169]	−0.139* [−0.244, −0.032]	0.099 [−0.009, 0.205]
Self-expression vs. Harmony	0.204*** [0.098, 0.305]	0.228*** [0.123, 0.328]	0.139* [0.032, 0.243]	−0.496*** [−0.573, −0.410]	−0.162** [−0.265, −0.055]
Self-interest vs. Commitment to others	0.370*** [0.273, 0.459]	0.336*** [0.237, 0.429]	0.325*** [0.225, 0.419]	−0.493*** [−0.571, −0.407]	−0.513*** [−0.589, −0.429]

*p < 0.05, **p < 0.01, ***p < 0.001.

## Discussion

### The Psychometric Properties of the SCS

A validation study of the SCS was conducted and the psychometric properties of the SCS were examined. As Bollen (1989b) pointed out, the replication on independent samples is the only way to check whether original associations are a sampling fluke or not. He also emphasized the necessity of such research, because despite the fact that replications are often considered very valuable, such studies appear far too seldom. This type of research therefore serves as a contribution to the cross-cultural examination of the I/C concept and its results are necessary for a deeper understanding of I/C across various cultures.

In summary, both questionnaires demonstrated limitations in their reliability and validity. These shortcomings could have stemmed from the lack of reliability and validity of the original versions (Singelis et al., 1995; Triandis and Gelfand, 1998; Bartoš, 2010; Vignoles et al., 2016) rather than our cross-cultural adaptation.

In more detail, despite that Czech version of the SCS showed satisfactory reliability in four subscales (the rest of the subscales were only slightly below the 0.70), similar correlations between subscales were observed and similar fit indices were obtained in comparison with the original study (Vignoles et al., 2016). Additionally, our study revealed several crucial psychometric shortcomings of the scale which suggest its insufficient validity and reliability. This might be to some extent a consequence of the psychometric properties of the original instrument.

Four main issues were identified. First, the reliability estimation with ipsative scores showed poor internal consistency suggesting that the SCS may be influenced by response biases. Second, the CFA results were unsatisfactory, and therefore cross-cultural comparisons using this questionnaire might be biased, non-invariant and invalid (Fischer and Karl, 2019). Both the original and Czech versions of the SCS have serious shortcomings in their factor structure, as suggested by, for example, the *CFI*.

Consequently, the third issue stemmed from an analysis of the modification indices, which revealed some cross-loadings. For example, the item “*You follow your personal goals even if they are very different from the goals of your family*” might saturate not only “Self-interest vs. Commitment to others,” but also “Self-containment vs. Connection to others,” “Self-direction vs. Receptiveness to influence” and “Self-expression vs. Harmony.” This finding seems logical, because a person who answers negatively to the mentioned item is probably not only more committed to others, but also leans toward harmony and receptiveness to an influence and is more connected to others. The presented analysis suggests that many items have similar cross-loadings. We believe that this is probably more likely caused by the poor theoretical background in the latent variables than vague and ambiguous item wording. Consequently, SCS factors are vaguely defined and lack divergent validity because they are based primarily on psychometric results. Therefore, even simply worded items (e.g., the item “*You always ask your family for advice before making a decision*”) have potential cross-loadings on other subscales. Furthermore, the semantic qualities of some factors seem to be quite similar (e.g., “Self-direction vs. Receptiveness to influence” and “Self-interest vs. Commitment to others”), and perhaps may be adequate to reduce the number of I/C factors. Although the process of validation of a multidimensional cross-cultural questionnaire like SCS is very tedious, it should not be limited just to the psychometric evaluation of factor structure, model fit, reliability, etc., but it should also be theoretically well grounded.

And four, despite that all directions of relationships between SCS and INDCOL were as expected, i.e., the dimensions of horizontal and vertical collectivism correlated with interdependent self, whereas the dimensions of horizontal and vertical individualism correlated with independent self, the correlation coefficients were mostly small or moderate. This suggests, that both scales probably measure slightly different and insufficiently related constructs The above-mentioned issues with the SCS lead us to questions about the I/C concept itself, because similar issues were observed in multiple previous studies (see below).

### General Issues of the I/C Concept

Research of I/C has been criticized by many scholars. Generally, there is no questionnaire in the literature measuring I/C that repeatedly meets the demanding requirements of cross-cultural research (i.e., CFA, MG-CFA, MI across different cultures, controlling for response bias, etc.). Many studies do not sufficiently conduct or report these important psychometric properties, and do not conduct any adequate multi-level analysis (Oyserman et al., 2002; Levine et al., 2003a,b; Chen and West, 2008; Cozma, 2011). Another relevant critique argues that the I/C research ignores or even lacks concurrent and discriminant validity of the scales (Oyserman et al., 2002; Levine et al., 2003b; Bresnahan et al., 2005; Schimmack et al., 2005). Furthermore, the conceptual unclarity of the I/C research is also often criticized (e.g., Oyserman et al., 2002; Voronov and Singer, 2002; Brewer and Chen, 2007; Oyserman and Lee, 2008).

As mentioned above, the original validation studies of INDCOL (Singelis et al., 1995; Triandis and Gelfand, 1998), SCS (Vignoles et al., 2016), VSM (Hofstede, 1994), SVS (Schwartz, 1992), and IISS (Lu and Gilmour, 2007) did not meet the minimum criteria of CFA or did not even perform such a procedure. Similar patterns can be found in other I/C questionnaires, for example in the W-M (Wagner and Moch, 1986), COS (Communal Orientation Scale; Clark et al., 1987), RISC (Relational-Interdependent Self-Construal; Cross et al., 2000), AICS (Auckland Individualism and Collectivism Scale; Shulruf et al., 2007) and ICIA scales (Collectivism Interpersonal Assessment Inventory; Matsumoto et al., 1997).

On the other hand, there are also several exceptions. For example, the Human Relations Questionnaire (HRQ; Noguchi, 2007) identifies three factors, namely the focus on others, helping others and self-focus, with satisfactory CFA indices: χ^2^(24) = 49.93, *GFI* = 0.973, *CFI* = 0.964, *RMSEA* = 0.052. Nevertheless, the author did not perform MG-CFA and MI analysis across the United States and Japanese versions, and its internal consistency was also not sufficient (α = 0.44-0.76). Three factors were also identified in the RIC scale (Relational, Individual and Collective Self-Aspects; Kashima and Hardie, 2000), namely relational, individual and collective self-aspects. The RIC scale showed satisfactory CFA fit indices [χ^2^(24) = 79.26, *CFI* = 0.94, *NFI* = 0.91]; however, the CFA was supported by a nine-item model (only 3 items per subscale), not the original 30-item model (*CFI* = 0.72).

Other examples can be the PVQ (Schwartz et al., 2001), or the Collective Self-Esteem Scale (CSES, Luhtanen and Crocker, 1992). The PVQ provided an acceptable MG-CFA but insufficient MI results (Anýžová, 2014), while the four-dimensional CSES reached close to satisfactory CFA results (*NFI* = 0.74–0.91, *TLI* = 0.84–0.92, *CFI* = 0.87–0.93). However, the CSES factor structure was not confirmed in the African American ethnic group, and therefore we can doubt its usability in a cross-cultural equivalence (Utsey and Constantine, 2006). Additionally, both questionnaires also focus primarily on different constructs that only possess a partial overlap with the I/C concept, i.e., cultural values in the case of PVQ and collective self-esteem in the case of CSES.

In this paper we conducted an attempt to validate an adaptation of a relatively new I/C scale on a Czech sample which is a sample not fitting into the group of West and East countries (such as the United States, England, Japan, China) that are studied the most often in the field. In this section we want to provide information about other similar research going beyond this dichotomy, both successful and unsuccessful ones. We omit adaptations without a CFA procedure or its adequate equivalent, which unfortunately represents the vast majority of studies (Chen and West, 2008). I/C scales were already successfully adapted for instance in Turkey (e.g., Li and Aksoy, 2006; Akın et al., 2010), Jordan, Lebanon, and Syria (e.g., Harb and Smith, 2008), Hong Kong and Ghana (e.g., Affum-Osei et al., 2019) or Switzerland and South Africa (results were satisfactory only for one of two used I/C scales; see Györkös et al., 2012).

Nevertheless, these successful attempts are relatively rare compared to the amount of studies that failed to do so. In spite of the fact that the authors themselves often interpreted the quality of the adaptations as satisfactory and part of studies is indeed methodologically and statistically sound, a deeper inspection reveals issues in the factor structure of the adapted scales. The adaptations usually did not yield satisfactory fit indices. In some cases, the number of factors and items were substantially changed compared to the original scales. Despite the fact that these model modifications and changes lead in some cases to the satisfactory fit indices of the “new” scales, this rather data-driven approach needs to be considered exploratory (see e.g., Bollen, 1989a; Byrne, 2010).

Similar psychometric problems with the adaptation of the I/C scale as in the current study were observed for instance in Poland (cf. Pilarska, 2011), Spain (cf. Gouveia et al., 2003), India (cf. Sivadas et al., 2008), Malaysia (cf. Miramontes, 2011; Ramley et al., 2020), Mexico (cf. Miramontes, 2011), Singapore (cf. Soh and Leong, 2002), Italy (cf. Bobbio and Sarrica, 2009; D’Amico and Scrima, 2015; Germani et al., 2020), France (cf. Gibas et al., 2016), Philippines (cf. Miramontes, 2011; Bernardo et al., 2012; Datu, 2014), Australia (cf. Freeman and Bordia, 2001; Miramontes, 2011), Portugal (cf. Gonçalves et al., 2017), Thailand (cf. Christopher et al., 2011) or Argentina (cf. Chiou, 2001).

The current, rather unsatisfactory results might have deeper causes than just the psychometric quality of the original SCS scale. Even though past studies assumed I/C being a stable cross-cultural construct with an ambition to categorize nations along the collectivistic and individualistic spectrum, these assumptions were not entirely confirmed. It seems that the relatively simplistic East-West dichotomy doesn’t truly exist (Matsumoto, 1999; Takano and Osaka, 1999, 2018; Heine et al., 2002; Oyserman et al., 2002; Levine et al., 2003a,b), and the I/C construct is far less stable than assumed (Yamagishi, 1988; Gardner et al., 1999; Oyserman and Lee, 2008). Consequently, some authors with respect to the previously mentioned shortcomings and critiques of I/C research came to the conclusion that the concept of I/C itself does not exist and suggest not using it in research (e.g., Levine et al., 2003a,b). Therefore, doubts about the validity of using I/C as a predictor of other constructs in current cross-cultural studies should be raised.

### Limitations and Future Directions

The results of our study are based on the unrepresentative sample gained through the non-probability convenience sampling method which resulted in various imbalances of demographic characteristics, especially in the overrepresentation of women, young participants and participants with a university education. An analysis performed on different populations might result in a different factor structure. However, I/C research usually validates the scales on samples of university students; for example, INDCOL (Singelis et al., 1995; Triandis and Gelfand, 1998) was created on the basis of such samples and SCS (Vignoles et al., 2016) used this population in the first phase of their validation study. Furthermore, despite the fact that sample size was considered satisfactory, SEM usually needs large samples and therefore is an *a priori* power analysis based on model simulations highly recommended (Hoyle, 2012; Little, 2013; Brown, 2015; Kline, 1998), which was not performed in the current study.

The future research should, in the first step, focus on a redefinition and reconceptualization of the I/C construct (e.g., Oyserman et al., 2002; Voronov and Singer, 2002; Brewer and Chen, 2007; Oyserman and Lee, 2008), while it is quite possible that such redefinition would not be universal for different cultural groups. Consequently, after the theoretical clarification of the I/C concept, the main aim of the future research should lie in a sounder methodological and statistical approach such as routinely using SEM techniques.

One of the possible ways to achieve this is the development of a new self-report instrument with satisfactory psychometric properties with the potential to be adapted in multiple cultures (Schimmack et al., 2005; Chen and West, 2008; Cozma, 2011). An important characteristic of this instrument would be its resistance to a reference-group effect (see Heine et al., 2002). Additionally, such an instrument would need to yield satisfactory results in repeated replications on independent samples from both the same and other cultures (Bollen, 1989b). Furthermore, adequate statistical approaches need to be used while comparing means across various cultural groups, such as MG-CFA with scalar measurement invariance (see Fischer and Karl, 2019). The research needs to be robust enough to cover the whole spectrum of variables that can potentially affect the level of I/C in order to reduce the cultural attribution fallacy (i.e., unpacking studies; see Matsumoto and Juang, 2013). Since the validation procedure usually does not end with one (un)successful validation study, but it represents an iterative process of bringing new evidence of validity and reliability the research in the field is far from concluded. However, we believe that without such an approach it is not possible to bring valid information about the real nature of I/C in culturally diverse populations via self-report scales.

The second possible approach to solve the current unsatisfactory situation in I/C research could lie in a shift from quantitative self-report questionnaires based on verbal responses to the usage of entirely different group of methods (e.g., Matsumoto, 1999; Bond, 2002; Fiske, 2002; Heine et al., 2002). For example, Talhelm et al. (2018) observed the differences in I/C with an observational design of the real-life behavior of participants; Partikova (2019) used interpretative phenomenological analysis of semi-structured interviews, and Hsu and Barker (2013) identified differences in I/C using the content analysis of TV advertisements. Furthermore, meta-analysis of other “cultural products” by Morling and Lamoreaux (2008) revealed higher effect sizes than meta-analyses performed on self-report scales. Another example can be found in the work of Klein et al. (2018) who created a new “WEIRDness score” (WEIRD: Western, educated, industrialized, rich and democratic; see Henrich et al., 2010) which might be included into multi-group statistical analysis as a predictor in a similar fashion as Hofstede’s dimensions. We believe that it might be possible to create a similar country-level index specifically related to the I/C. Maybe not on the basis of self-report questionnaire data, but rather from an in-depth qualitative analysis of several indicators and consequent inter-rater agreement of experts from various cultures.

## Data Availability Statement

The raw data supporting the conclusions of this article will be made available by the authors, without undue reservation.

## Ethics Statement

The studies involving human participants were reviewed and approved by The Research Ethics Committee of Masaryk University (Ref. No.: EKV-2018-011, Proposal No.: 0257/2018). Written informed consent for participation was not required for this study in accordance with the national legislation and the institutional requirements. Written informed consent was implied via completion of the questionnaires.

## Author Contributions

DL contributed to data collection, article drafting, and data analysis. JČ contributed to questionnaire adaptation, data collection, and article drafting. TU contributed to article revisions and data analysis. All authors contributed to the article and approved the submitted version.

## Conflict of Interest

The authors declare that the research was conducted in the absence of any commercial or financial relationships that could be construed as a potential conflict of interest.

## References

[B1] Affum-Osei E. Aboagye M. O. Antwi C. O. Asante E. A. (2019). Validating the Auckland individualism–collectivism scale (AICS): testing factor structure and measurement invariance in Hong Kong and Ghanaian Samples. *Psychol. Stud.* 64 187–199. 10.1007/s12646-019-00494-2

[B2] Akın A. Eroğlu Y. Kayış A. R. Satıcı S. A. (2010). The validity and reliability of the Turkish version of the relational-interdependent self-construal scale. *Proc. Soc. Behav. Sci.* 5 579–584. 10.1016/j.sbspro.2010.07.145

[B3] Anýžová P. (2014). SrovnatelnostSchwartzovyhodnotovéškály v mezinárodníchdatech [The Comparability of Schwartz’s Human Values Scale in International Data]. *Czech. Sociol. Rev.* 50 547–580. 10.13060/00380288.2014.50.4.108

[B4] Bartoš F. (2010). Individualismus a kolektivismus v česképopulaci a jejichsouvislosti s narcismem [Individualism and Collectivism of the Czech Population and their Relation to Narcissism]. *Sociol. Slovak Sociol. Rev.* 42 134–161.

[B5] Bašnáková J. Brezina I. Masaryk R. (2016). Dimensions of culture: the case of Slovakia as an outlier in Hofstede’s research. *Cesk. Psychol.* 60 13–25.

[B6] Bernardo A. B. I. Lising R. L. S. Shulruf B. (2012). Validity of two language versions of the Auckland individualism and collectivism scale with Filipino-english bilinguals. *Psychol. Stud.* 58 33–37. 10.1007/s12646-012-0172-8

[B7] Blodgett J. G. Bakir A. Rose G. M. (2008). A test of the validity of Hofstede’s cultural framework. *J. Consum. Mark.* 25 339–349. 10.1108/07363760810902477

[B8] Bobbio A. Sarrica M. (2009). Horizontal and vertical individualism and collectivism: an Italian adaptation of Singelis et al.’s scale and its relations with conflict management and leadership styles. *TPM Test. Psychom. Methodol. Appl. Psychol.* 16 209–226.

[B9] Bollen K. A. (1989b). *Structural Equations with Latent Variables.* New York, NY: John Wiley & Sons.

[B10] Bollen K. A. (1989a). A new incremental fix index for general structural equation models. *Sociol. Methods. Res.* 17 303–316. 10.1177/0049124189017003004

[B11] Bond M. (2002). Reclaiming the individual from Hofstede’s ecological analysis: a 20-year odyssey: comment on Oyserman et al. (2002). *Psychol. Bull.* 128 73–77. 10.1037/0033-2909.128.1.73 11843548

[B12] Bresnahan M. J. Levine T. R. Shearman S. M. Lee S. Y. Park C.-Y. Kiyomiya T. (2005). A multimethod multitrait validity assessment of self-construal in Japan, Korea, and the United States. *Hum. Commun. Res.* 31 33–59. 10.1111/j.1468-2958.2005.tb00864.x

[B13] Brewer M. B. Chen Y.-R. (2007). Where (Who) are collectives in collectivism? Toward conceptual clarification of individualism and collectivism. *Psychol. Rev.* 114 133–151. 10.1037/0033-295x.114.1.133 17227184

[B14] Brown T. A. (2015). *Confirmatory Factor Analysis for Applied Research.* 2nd Edn. New York, NY: The Guilford Press.

[B15] Byrne B. M. (2010). *Structural Equation Modeling with Amos: Basic Concepts, Applications and Programming.* London: Routledge/Taylor & Francis Group.

[B16] Carlson K. D. Herdman A. O. (2010). Understanding the impact of convergent validity on research results. *Organ. Res. Methods* 15 17–32. 10.1177/1094428110392383

[B17] Čeněk J. (2015). Cultural dimension of individualism and collectivism and its perceptual and cognitive correlates in cross-cultural research. *J. Educ. Cult. Soc.* 2015 210–225. 10.15503/jecs20152.210.225

[B18] Čeněk J. Urbánek T. (2019). The adaptation and equivalence of test methods: an inspiration for psychological assessment of minorities in the Czech Republic. *Cesk. Psychol.* 63 42–54.

[B19] Chen F. F. West S. G. (2008). Measuring individualism and collectivism: the importance of considering differential components, reference groups, and measurement invariance. *J. Res. Pers.* 42 259–294. 10.1016/j.jrp.2007.05.006

[B20] Chiou J. S. (2001). Horizontal and vertical individualism and collectivism among college students in the United States, Taiwan, and Argentina. *J. Soc. Psychol.* 141 667–678. 10.1080/00224540109600580 11758044

[B21] Christopher M. S. Norris P. D’Souza J. B. Tiernan K. A. (2011). A test of the multidimensionality of the self-construal scale in Thailand and the United States. *J. Cross. Cult. Psychol.* 43 758–773. 10.1177/0022022111406119

[B22] Clark M. Ouellette R. Powell M. Milberg S. (1987). Recipient’s mood, relationship type, and helping. *J. Pers. Soc. Psychol.* 53 94–103. 10.1037/0022-3514.53.1.94 3612495

[B23] Cozma I. (2011). How are individualism and collectivism measured? *Rom. J. Appl. Psychol.* 13 11–17.

[B24] Cross S. E. Bacon P. L. Morris M. L. (2000). The relational-interdependent self-construal and relationships. *J. Pers. Soc. Psychol.* 78 791–808. 10.1037/0022-3514.78.4.791 10794381

[B25] Datu J. A. D. (2014). Validating the revised self-construal scale in the Philippines. *Curr. Psychol.* 34 626–633. 10.1007/s12144-014-9275-9

[B26] Dumetz J. Gáboriková E. (2017). The Czech and Slovak Republics: a cross-cultural comparison. *Marketing Sci. Inspir.* 11 2–13.

[B27] D’Amico A. Scrima F. (2015). The Italian validation of Singelis’s self-construal scale (SCS): a short 10-item version shows improved psychometric properties. *Curr. Psychol.* 35 159–168. 10.1007/s12144-015-9378-y

[B28] Finney S. J. DiStefano C. (2013). “Nonnormal and categorical data in structural equation modelling,” in *Quantitative Methods in Education and the Behavioral Sciences: Issues, Research, and Teaching. Structural Equation Modeling: A Second Course*, eds Hancock G. R. Mueller R. O. (Charlotte: IAP Information Age Publishing), 439–492.

[B29] Fischer R. Karl J. A. (2019). A primer to (Cross-Cultural) multi-group invariance testing possibilities in R. *Front. Psychol.* 10:1507. 10.3389/fpsyg.2019.01507 31379641 PMC6657455

[B30] Fischer R. Milfont T. L. (2010). Standardization in psychology research. *Int. J. Psychol. Res.* 3 88–96. 10.21500/20112084.852

[B31] Fischer R. (2004). Standardization to account for cross-cultural response bias: a classification of score adjustment procedures and review of research in JCCP. *J. Cross. Cult. Psychol.* 35 263–282. 10.1177/0022022104264122

[B32] Fiske A. P. (2002). Using individualism and collectivism to compare cultures–a critique of the validity and measurement of the constructs: comment on Oyserman et al. (2002). *Psychol. Bull.* 128 78–88. 10.1037/0033-2909.128.1.78 11843549

[B33] Fornell C. Larcker D. (1981). Evaluating structural equation models with unobservable variables and measurement error. *J. Mark. Res.* 18 39–50. 10.2307/3151312

[B34] Freeman M. A. Bordia P. (2001). Assessing alternative models of individualism and collectivism: a confirmatory factor analysis. *Eur. J. Pers.* 15 105–121. 10.1002/per.398

[B35] Gardner W. Gabriel S. Lee A. (1999). “I” value freedom, but “we” value relationships: self-construal priming mirrors cultural differences in judgment. *Psychol. Sci.* 10 321–326. 10.1111/1467-9280.00162

[B36] Germani A. Delvecchio E. Li J. B. Mazzeschi C. (2020). The horizontal and vertical individualism and collectivism scale: early evidence on validation in an Italian sample. *J. Child Fam. Stud.* 29 904–911. 10.1007/s10826-019-01571-w

[B37] Gibas D. Giraud T. Le Conte J. Rubens L. Martin J. C. Isableu B. (2016). Attempt to validate the self-construal scale in French: systematic approach and model limitation. *Eur. Rev. Appl. Psychol.* 66 85–93. 10.1016/j.erap.2016.02.001

[B38] Gonçalves G. Sousa C. Santos J. Gomes A. Santa-Rita A. Hipólito S. (2017). Psychometric properties of the Portuguese version of the relational interdependent self–construal scale. *Psicol. Rev. Associação Portuguesa Psicol.* 31 121–136. 10.17575/rpsicol.v31i2.1264

[B39] Gouveia V. V. Clemente M. Espinosa P. (2003). The horizontal and vertical attributes of individualism and collectivism in a Spanish population. *J. Soc. Psychol.* 143 43–63. 10.1080/00224540309598430 12617346

[B40] Györkös C. Becker J. Massoudi K. Antonietti J.-P. Pocnet C. de Bruin G. P. (2012). Comparing the horizontal and vertical individualism and collectivism scale and the Auckland individualism and collectivism scale in two cultures. *Cross. Cult. Res.* 47 310–331. 10.1177/1069397112470371

[B41] Hair J. F. Babin B. J. Anderson R. E. Black W. C. (2018). *Multivariate Data Analysis*, 8th Edn. Boston, MA: Cengage.

[B42] Harb C. Smith P. B. (2008). Self-construals across cultures. *J. Cross. Cult. Psychol.* 39 178–197. 10.1177/0022022107313861

[B43] Heine S. J. Lehman D. R. Peng K. Greenholtz J. (2002). What’s wrong with cross-cultural comparisons of subjective Likert scales? The reference-group effect. *J. Pers. Soc. Psychol.* 82 903–918. 10.1037/0022-3514.82.6.903 12051579

[B44] Henrich J. Heine S. J. Norenzayan A. (2010). The weirdest people in the world? *Behav. Brain. Sci.* 33 61–83. 10.1017/S0140525X0999152X 20550733

[B45] Hnilica K. Rendlová M. Bariekzahyová T. Hnilica M. (2006). Životní standard, individualistickéhodnoty a spokojenost se životem [Life standard, individualistic values, and life satisfaction]. *Cesk. Psychol.* 50 201–217.

[B46] Hofstede G. Hofstede G. J. Minkov M. (2010). *Cultures and Organizations: Software of the Mind*, Third Edn. New York, NY: McGraf-Hill.

[B47] Hofstede G. (1983). The cultural relativity of organizational practices and theories. *J. Int. Bus. Stud.* 14 75–89. 10.1057/palgrave.jibs.8490867

[B48] Hofstede G. (1994). *Value Survey Module 1994 Manual.* Maastricht: Institute for Research on Intercultural Cooperation.

[B49] Hooper D. Coughlan J. Mullen M. R. (2008). Structural equation modelling: guidelines for determining model fit. *Electron. J. Bus. Res. Methods* 6 53–60. 10.21427/D7CF7R

[B50] Hoyle R. H. (2012). *Handbook of Structural Equation Modeling.* New York, NY: The Guilford Press.

[B51] Hu L. Bentler P. M. (1999). Cutoff criteria for fit indexes in covariance structure analysis: conventional criteria versus new alternatives. *Struct. Equ. Model.* 6 1–55. 10.1080/10705519909540118

[B52] Hsu S. Y. Barker G. G. (2013). Individualism and collectivism in Chinese and American television advertising. *Int. Commun. Gaz.* 75 695–714. 10.1177/1748048513482543

[B53] Jorgensen T. D. Pornprasertmanit S. Schoemann A. M. Rosseel Y. (2018). *semTools: Useful Tools for Structural Equation Modeling*. R Package Version 0.5-3. Available online at: https://CRAN.R-project.org/package=semTools

[B54] Kashima E. Hardie E. (2000). The development and validation of the relational, individual, and collective self-aspects (RIC) scale. *Asian J. Soc. Psychol.* 3 19–48. 10.1111/1467-839X.00053

[B55] Klein R. A. Vianello M. Hasselman F. Adams B. G. Adams R. B. Alper S. (2018). Many labs 2: investigating variation in replicability across samples and settings. *Adv. Methods Pract. Psychol. Sci.* 1 443–490. 10.1177/2515245918810225

[B56] Kline R. B. (1998). *Principles and Practice of Structural Equation Modeling*, 4th Edn. New York, NY: The Guilford Press.

[B57] Kolman L. Noorderhaven N. Hofstede G. Dienes E. (2003). Cross−cultural differences in Central Europe. *J. Manag. Psychol.* 18 76–88. 10.1108/02683940310459600

[B58] Korkmaz S. Goksuluk D. Zararsiz G. (2014). MVN: an R package for assessing multivariate normality. *R J.* 6 151–162. 10.32614/RJ-2014-031

[B59] Lacko D. Čeněk J. (2020). “Factor structure of two individualism and collectivism scales,” in *PhD Existence 10; Česko-slovenskápsychologickákonference (nejen) Pro Doktorandy a o Doktorandech*, eds Maierová E. Viktorová L. Dolejš M. Dominik T. (Olomouc: Palacký University), 18–24.

[B60] Lacko D. Šašinka Č Čeněk J. Stachoň Z. Lu W. (2020). Cross-cultural differences in cognitive style, individualism/collectivism and map reading between Central European and East Asian University students. *Stud. Psychol. (Bratisl)* 62 23–43. 10.31577/sp.2020.01.789

[B61] Levine T. R. Bresnahan M. J. Park H. S. Lapinski M. K. Wittenbaum G. Shearman S. (2003b). Self-construal scales lack validity. *Hum. Commun. Res.* 29 210–252. 10.1111/j.1468-2958.2003.tb00837.x

[B62] Levine T. R. Bresnahan M. J. Park H. S. Lapinski M. K. Lee T. S. Lee D. W. (2003a). The (In)validity of self-construal scales revisited. *Hum. Commun. Res.* 29 291–308. 10.1111/j.1468-2958.2003.tb00840.x

[B63] Li C. (2016). Confirmatory factor analysis with ordinal data: comparing robust maximum likelihood and diagonally weighted least squares. *Behav. Res. Methods* 48 936–949. 10.3758/s13428-015-0619-7 26174714

[B64] Li F. Aksoy L. (2006). Dimensionality of individualism–collectivism and measurement equivalence of Triandis and Gelfand’s scale. *J. Bus. Psychol.* 21 313–329. 10.1007/s10869-006-9031-8

[B65] Little T. D. (2013). *Longitudinal Structural Equation Modeling.* New York, NY: The Guilford Press.

[B66] Lu L. Gilmour R. (2007). Developing a new measure of independent and interdependent views of the self. *J. Res. Pers.* 41 249–257. 10.1016/j.jrp.2006.09.005

[B67] Luhtanen R. Crocker J. (1992). A collective self-esteem scale: self-evaluation of one’s social identity. *Pers. Soc. Psychol. Bull.* 18 302–318. 10.1177/0146167292183006

[B68] Markus H. Kitayama S. (1991). Culture and the self: implications for cognition, emotion, and motivation. *Psychol. Rev.* 98 224–253. 10.1037/0033-295X.98.2

[B69] Martinkova P. Drabinova A. (2018). ShinyItemAnalysis for teaching psychometrics and to enforce routine analysis of educational tests. *R J.* 10 503–515. 10.32614/RJ-2018-074

[B70] Matsumoto D. Weissman M. Preston K. Brown B. Kupperbush C. (1997). Context-specific measurement of individualism-collectivism on the individual level: the individualism-collectivism interpersonal assessment inventory. *J. Cross. Cult. Psychol.* 28 743–767. 10.1177/0022022197286006

[B71] Matsumoto D. (1999). Culture and self: an empirical assessment of Markus and Kitayama’s theory of independent and interdependent self-construals. *Asian J. Soc. Psychol.* 2 289–310. 10.1111/1467-839x.00042

[B72] Matsumoto D. Juang L. (2013). *Culture & Psychology.* Belmont: Wadsworth Publishing.

[B73] Morling B. Lamoreaux M. (2008). Measuring culture outside the head: a meta-analysis of individualism-collectivism in cultural products. *Pers. Soc. Psychol. Rev.* 12 199–221. 10.1177/1088868308318260 18544712

[B74] McSweeney B. (2002). Hofstede’s model of national cultural differences and their consequences: A triumph of faith -a failure of analysis. *Hum. Relat.* 55 89–118. 10.1177/0018726702551004

[B75] Miramontes L. G. (2011). *The Structure and Measurement of Self-Construals: A Cross-Cultural Study of the Self-Construal Scale.* dissertation thesis. Washington, DC: Washington State University.

[B76] Noguchi K. (2007). Examination of the content of individualism/collectivism scales in the cultural comparisons of the USA and Japan. *Asian J. Soc. Psychol.* 10 131–144. 10.1111/j.1467-839X.2007.00220.x

[B77] Oyserman D. Lee S. (2008). Does culture influence what and how we think? Effects of priming individualism and collectivism. *Psychol. Bull.* 134 311–342. 10.1037/0033-2909.134.2.311 18298274

[B78] Oyserman D. Coon H. Kemmelmeier M. (2002). Rethinking individualism and collectivism: evaluation of theoretical assumptions and meta-analyses. *Psychol. Bull.* 128 3–72. 10.1037/0033-2909.128.1.3 11843547

[B79] Partikova V. (2019). Exploring the self-perception of kung fu teachers. An interpretative phenomenological analysis. *Eur. J. Sport Soc.* 16 247–267. 10.1080/16138171.2019.1661143

[B80] Pilarska A. (2011). PolskaadaptacjaSkaliKonstruktówJa [Polish adaptation of Self-Construal Scale]. *Studia Psychol.* 49 21–34. 10.2478/v10167-011-0002-y

[B81] R Core Team (2020). *R: A Language and Environment for Statistical Computing.* Vienna: R Foundation for Statistical Computing.

[B82] Ramley F. Karnilowicz W. Yasin M. A. S. M. Balqis S. Nor M. (2020). An analysis of cross-cultural equivalence of self-construal scale in Malaysia. *Int. J. Adv. Appl. Sci.* 7 91–98. 10.21833/ijaas.2020.02.013

[B83] Revelle W. (2020). *psych: Procedures for Psychological, Psychometric, and Personality Research*. R Package Version 2.1.3. Evanston, IL: Northwestern University.

[B84] Řeháková B. (2006). Měřeníhodnotovýchorientacímetodouhodnotovýchportrétů S. H. Schwartze [Measuring Value Orientations with the Use of S. H. Schwartz’s Value Portraits]. *Czech. Sociol. Rev.* 42 107–128. 10.13060/00380288.2006.42.1.07

[B85] Rosseel Y. (2012). lavaan: an R package for structural equation modeling. *J. Stat. Softw.* 48 1–36. 10.18637/jss.v048.i02

[B86] Santos H. C. Varnum M. E. W. Grossmann I. (2017). Global increases in individualism. *Psychol. Sci.* 28 1228–1239. 10.1177/0956797617700622 28703638

[B87] Schimmack U. Oishi S. Diener E. (2005). Individualism: a valid and important dimension of cultural differences between nations. *Pers. Soc. Psychol. Rev.* 9 17–31. 10.1207/s15327957pspr0901_2 15745862

[B88] Schwartz S. (1992). Universals in the content and structure of values: theoretical advances and empirical tests in 20 Countries. *Adv. Exp. Soc. Psychol.* 22 1–65. 10.1016/S0065-2601(08)60281-6

[B89] Schwartz S. Melech G. Lehrnami A. Burgess S. Harris M. Owens V. (2001). Extending the cross-cultural validity of the theory of basic human values with a different method of measurement. *J. Cross. Cult. Psychol.* 32 519–542. 10.1177/0022022101032005001

[B90] Shulruf B. Hattie J. Dixon R. (2007). Development of a new measurement tool for individualism and collectivism. *J. Psychoeduc. Assess.* 25 385–401. 10.1177/0734282906298992

[B91] Singelis T. Triandis H. Bhawuk D. Gelfand M. (1995). Horizontal and vertical dimensions of individualism and collectivism: a theoretical and measurement refinement. *Cross Cult. Res.* 29 240–275. 10.1177/106939719502900302

[B92] Sivadas E. Bruvold N. T. Nelson M. R. (2008). A reduced version of the horizontal and vertical individualism and collectivism scale: a four-country assessment. *J. Bus. Res.* 61 201–210. 10.1016/j.jbusres.2007.06.016

[B93] Smith P. Fischer R. Vignoles V. Bond M. (2013). *Understanding Social Psychology Across Cultures: Engaging With Others in a Changing World.* London: Sage.

[B94] Smith P. Vignoles V. Becker M. Owe E. Easterbrook M. Brown R. (2016). Individual and culture-level components of survey response styles: a multi-level analysis using cultural models of selfhood. *Int. J. Psychol.* 51 453–463. 10.1002/ijop.12293 27374874

[B95] Soh S. Leong F. T. L. (2002). Validity of vertical and horizontal individualism and collectivism in Singapore. *J. Cross. Cult. Psychol.* 33 3–15. 10.1177/0022022102033001001

[B96] Talhelm T. Zhang X. Oishi S. (2018). Moving chairs in starbucks: observational studies find rice-wheat cultural differences in daily life in China. *Sci. Adv.* 4:eaa8469. 10.1126/sciadv.aap8469 29707634 PMC5916507

[B97] Takano Y. Osaka E. (1999). An Unsupported common view: comparing Japan and the US on individualism/collectivism. *Asian J. Soc. Psychol.* 2 311–341. 10.1111/1467-839X.00043

[B98] Takano Y. Osaka E. (2018). Comparing Japan and the United States on individualism/collectivism: a follow-up review. *Asian J. Soc. Psychol.* 21 301–316. 10.1111/ajsp.12322

[B99] Triandis H. Gelfand M. (1998). Converging measurement of horizontal and vertical individualism and collectivism. *J. Pers. Soc. Psychol.* 74 118–128. 10.1037/0022-3514.74.1.118

[B100] Uskul A. K. Oyserman D. (2006). “Question comprehension and response: implications of individualism and collectivism,” in *National Culture and Groups*, Vol. 9 ed. Chen Y.-R. (Bingley: Emerald Publishing), 173–201. 10.1016/S1534-0856(06)09008-6

[B101] Utsey S. Constantine M. (2006). A confirmatory test of the underlying factor structure of scores on the collective self-esteem scale in two independent samples of black Americans. *J. Pers. Assess.* 86 172–179. 10.1207/s15327752jpa8602_06 16599791

[B102] van de Vijver F. Hambleton R. (1996). Translating tests: some practical guidelines. *Eur. Psychol.* 1 89–99. 10.1027/1016-9040.1.2.89

[B103] van de Vijver F. Leung K. (2001). Personality in cultural context: methodological issues. *J. Pers.* 69 1007–1031. 10.1111/1467-6494.696173 11767816

[B104] van de Vijver F. Tanzer N. K. (1997). Bias and equivalence in cross-cultural assessment: an overview. *Eur. Rev. of App. Psychol.* 47 263–280. 10.1016/j.erap.2003.12.004

[B105] Varnum M. Grossmann I. Kitayama S. Nisbett R. (2010). The origin of cultural differences in cognition: evidence for the social orientation hypothesis. *Curr. Dir. Psychol. Sci.* 19 9–13. 10.1177/0963721409359301 20234850 PMC2838233

[B106] Vignoles V. Owe E. Becker M. Smith P. Easterbrook M. Brown R. (2016). Beyond the ‘East-West’ dichotomy: global variation of cultural models of selfhood. *J. Exp. Psychol. Gen.* 145 966–1000. 10.1037/xge0000175 27359126

[B107] Voronov M. Singer J. (2002). The myth of individualism-collectivism: a critical review. *J. Soc. Psychol.* 142 461–480. 10.1080/00224540209603912 12153123

[B108] Wagner J. Moch K. (1986). Individualism-collectivism: concept and measure. *Group Organ. Stud.* 11 280–304. 10.1177/105960118601100309

[B109] Welkenhuysen-Gybels J. Billiet J. Cambré B. (2003). Adjustment for acquiescence in the assessment of the construct equivalence of Likert type score items. *J. Cross. Cult. Psychol.* 34 702–722. 10.1177/0022022103257070

[B110] Yamagishi T. (1988). The provision of a sanctioning system in the United States and Japan. *Soc. Psychol. Q.* 51 265–271. 10.2307/2786924

