# Supplementary material for: Psychometric Properties of the Independent and Interdependent Self-Construal Questionnaire: Evidence From the Czech Republic
Source: Front Psychol. 2021 Jun 3;12:564011. doi: 10.3389/fpsyg.2021.564011 (PMC8209258; doi:10.3389/fpsyg.2021.564011)
Supplement: Supplementary file 1 [file Table_1.docx]

# Appendix I

Appendix I: Standardized factor loadings for the Czech version of Self-Construal Scale (SCS):

| *Item* | *Dimension* | *SCS CZ λ* | *SCS orig λ* |
| --- | --- | --- | --- |
|  | **Difference vs. Similarity** |  |  |
| 1 | ENG: You like being different from other people.  CZE: Rád/a se od ostatních lidí odlišujete. | .645 | .569 |
| 2 | ENG: You see yourself as unique and different from others.  CZE: Připadáte si jedinečný/á a jiný/á než ostatní. | .550 | .492 |
| 3 | ENG: You like it when people notice you in a group.  CZE: Rád/a mezi ostatními ve skupině vyčníváte. | .662 | .265 |
| 4 | ENG: Being different from others makes you feel uncomfortable.  CZE: Cítíte se nepříjemně, když se od ostatních odlišujete. | - .700 | - .460 |
| 5 | ENG: You try to avoid being noticeably different from others.  CZE: Snažíte se od ostatních výrazně nelišit. | - .781 | - .342 |
| 6 | ENG: Being praised in front of others makes you feel uncomfortable.  CZE: Cítíte se nepříjemně, když vás někdo chválí před ostatními. | - .294 | - .261 |
|  | **Self-containment vs. Connection to others** |  |  |
| 7 | ENG: Your happiness is unrelated to the happiness of your family.  CZE: Vaše štěstí je nezávislé na štěstí vaší rodiny. | .568 | .450 |
| 8 | ENG: When you talk about yourself, you don’t say very much about your family.  CZE: Když s někým mluvíte o sobě, většinou nemluvíte příliš o vaší rodině. | .480 | .244 |
| 9 | ENG: If someone insults a friend, you rarely feel insulted yourself.  CZE: Většinou se neurazíte, když někdo urazí vašeho kamaráda. | .347 | .178 |
| 10 | ENG: If someone in your family is sad, you feel the sadness as if it were your own.  CZE: Když je někdo ve vaší rodině smutný, pociťujete tento smutek jako váš vlastní. | - .661 | - .554 |
| 11 | ENG: When someone in your family achieves something, you feel proud as if you had achieved something yourself.  CZE: Když někdo ve vaší rodině dosáhne úspěchu, pociťujete stejnou hrdost, jako by se jednalo o váš vlastní úspěch. | - .627 | - .485 |
| 12 | ENG: Your happiness depends on the happiness of your friends.  CZE: Dokážete být šťastný/á, jen když jsou šťastni i vaši přátelé. | - .518 | - .159 |
|  | **Self-direction vs. Receptivness to influence** |  |  |
| 13 | ENG: You prefer to do what you want without letting your family influence you.  CZE: Upřednostňujete dělat si věci po svém a nenecháte se při tom ovlivnit svou rodinou. | .674 | .595 |
| 14 | ENG: You make decisions about your life on your own.  CZE: O svém životě rozhodujete podle sebe. | .652 | .430 |
| 15 | ENG: You always ask your family for advice before making a decision.  CZE: Před tím, než se rozhodnete, vždy se poradíte se svou rodinou. | - .508 | - .510 |
| 16 | ENG: Other people have a great influence over the choices you make.  CZE: Ostatní lidé značně ovlivňují to, jak se rozhodnete. | - .546 | - .317 |
|  | **Self-reliance vs. Dependence on others** |  |  |
| 17 | ENG: You prefer to rely completely on yourself rather than depend on others.  CZE: Raději se spoléháte výhradně na sebe, než abyste se musel/a spoléhat na druhé. | .684 | .586 |
| 18 | ENG: You try to avoid being reliant on others.  CZE: Snažíte se být nezávislý/á na ostatních. | .639 | .468 |
| 19 | ENG: You prefer to ask other people for help rather than rely on yourself.  CZE: Raději žádáte ostatní o pomoc, než abyste se spoléhal/a sám/sama na sebe. | - .690 | - .506 |
| 20 | ENG: You feel uncomfortable in situations where you have to rely only on yourself.  CZE: Cítíte se nepříjemně v situacích, kdy se musíte spoléhat jen sám/sama na sebe. | - .702 | - .499 |
|  | **Consistency vs. Variability** |  |  |
| 21 | ENG: You behave in the same way even when you are with different groups of people.  CZE: Chováte se stejně, i když jste s různými skupinami lidí. | .729 | .611 |
| 22 | ENG: You always see yourself in the same way even when you are with different people.  CZE: Váš pohled na sebe sama je stejný bez ohledu na to, s jakými lidmi zrovna jste. | .732 | .547 |
| 23 | ENG: You behave the same way at home and in public.  CZE: Doma i na veřejnosti se chováte stejně. | .704 | .581 |
| 24 | ENG: You act very differently at home compared to how you act in public.  CZE: Doma se chováte velmi odlišně od toho, jak se se chováte na veřejnosti. | - .648 | - .546 |
| 25 | ENG: You see yourself differently in different social environments.  CZE: Váš pohled na sebe sama se mění podle toho, mezi jakými lidmi se zrovna nacházíte. | - .697 | - .474 |
| 26 | ENG: You behave differently when you are with different groups of people.  CZE: Když jste s různými skupinami lidí, chováte se rozdílně. | - .724 | - .518 |
|  | **Self-Expression vs. Harmony** |  |  |
| 27 | ENG: You prefer to say what you are thinking, even if it is inappropriate for the situation.  CZE: Říkáte to, co si myslíte, i když je to v dané situaci nevhodné. | .469 | .438 |
| 28 | ENG: You show your inner feelings even if it disturbs the harmony in your family.  CZE: Projevujete své pocity i za cenu narušení rodinné pohody. | .493 | .473 |
| 29 | ENG: You are comfortable expressing disagreement with friends.  CZE: Nedělá vám problém, když nesouhlasíte se svými přáteli. | .522 | .314 |
| 30 | ENG: You try to adapt to people around you, even if it means hiding your inner feelings.  CZE: Snažíte se lidem ve vašem okolí přizpůsobit i za cenu toho, že skrýváte to, jak se skutečně cítíte. | - .612 | - .315 |
| 31 | ENG: You feel uncomfortable when you express disagreement with members of your family  CZE: Vyjádřit nesouhlas s vaší rodinou je pro vás nepříjemné. | - .534 | - .299 |
| 32 | ENG: You try to maintain harmony among the people around you.  CZE: Snažíte se udržovat pohodu mezi lidmi okolo vás. | - .287 | - .229 |
|  | **Self-interest vs. Commitment to others** |  |  |
| 33 | ENG: You value personal achievements more than good relations with the people close to you.  CZE: Osobních úspěchů si vážíte více než dobrých vztahů s lidmi, kteří jsou vám blízcí. | .566 | .473 |
| 34 | ENG: Your own success is very important to you, even if it disrupts your friendships.  CZE: Váš vlastní úspěch je pro vás velmi důležitý, a to i pokud by mohl narušit vaše přátelství s druhými. | .585 | .392 |
| 35 | ENG: You follow your personal goals even if they are very different from the goals of your family.  CZE: Jdete si za vlastními cíli, i pokud se výrazně liší od cílů vaší rodiny. | .628 | .523 |
| 36 | ENG: You value good relations with the people close to you more than your personal achievements.  CZE: Dobrých vztahů s vašimi blízkými si ceníte víc než svých osobních úspěchů. | - .584 | - .407 |
| 37 | ENG: You always put your family first, even if it means giving up your personal goals.  CZE: Rodina je u vás vždy na prvním místě, i kdyby to znamenalo vzdát se vlastních cílů. | - .627 | - .490 |
| 38 | ENG: You are more concerned with your friends‘ happiness than your own success.  CZE: Staráte se o štěstí vašich přátel víc než o vlastní úspěch. | - .493 | - .224 |

λ = Factor loading.
