# Supplementary material for: Psychometric Properties of the Independent and Interdependent Self-Construal Questionnaire: Evidence From the Czech Republic
Source: Front Psychol. 2021 Jun 3;12:564011. doi: 10.3389/fpsyg.2021.564011 (PMC8209258; doi:10.3389/fpsyg.2021.564011)
Supplement: Supplementary file 2 [file Table_2.DOCX]

# Appendix II

Appendix II: Traditional item analysis for the Czech version of Self-Construal Scale (SCS):

| *Item* | *Me* | *IQR* | *M* | *SD* | *Alpha drop* | *ULI* | *RIT* | *RIR* |
| --- | --- | --- | --- | --- | --- | --- | --- | --- |
| 1 | 6 | 3 | 5.56 | 2.03 | .66 | .15 | .65 | .51 |
| 2 | 6 | 2 | 5.79 | 2.07 | .70 | .29 | .58 | .35 |
| 3 | 5 | 5 | 4.72 | 2.36 | .69 | .13 | .55 | .40 |
| 4 | 4 | 4 | 3.94 | 2.07 | .62 | .35 | .76 | .58 |
| 5 | 3 | 3 | 3.82 | 1.90 | .65 | .29 | .71 | .52 |
| 6 | 5 | 4 | 5.22 | 2.40 | .70 | .30 | .59 | .35 |
| 7 | 4 | 3 | 4.31 | 2.19 | .47 | .13 | .67 | .43 |
| 8 | 5 | 4 | 4.63 | 2.33 | .52 | .15 | .62 | .34 |
| 9 | 4 | 2.75 | 3.99 | 2.11 | .57 | .09 | .48 | .22 |
| 10 | 6 | 3 | 5.82 | 2.16 | .51 | .05 | .57 | .40 |
| 11 | 7 | 2.75 | 6.57 | 2.10 | .54 | .06 | .49 | .28 |
| 12 | 5 | 3.75 | 4.85 | 2.09 | .56 | .15 | .58 | .27 |
| 13 | 6 | 2 | 6.13 | 1.83 | .27 | .27 | .78 | .54 |
| 14 | 7 | 2 | 6.84 | 1.71 | .51 | .53 | .72 | .31 |
| 15 | 4 | 4 | 4.39 | 2.28 | .51 | .30 | .61 | .27 |
| 16 | 4 | 3 | 4.48 | 2.02 | .53 | .11 | .47 | 24 |
| 17 | 7 | 3 | 6.50 | 1.94 | .63 | .46 | .79 | .60 |
| 18 | 7 | 2 | 6.84 | 1.73 | .68 | .46 | .73 | .51 |
| 19 | 3 | 2 | 3.21 | 1.92 | .66 | .63 | .78 | .55 |
| 20 | 3 | 3 | 3.70 | 2.16 | .72 | .44 | 68 | .44 |
| 21 | 4 | 4 | 4.79 | 2.05 | .59 | .06 | .53 | .39 |
| 22 | 5 | 4 | 5.54 | 2.27 | .54 | .28 | .72 | .47 |
| 23 | 5 | 4 | 5.04 | 2.27 | .58 | .13 | .58 | .38 |
| 24 | 4 | 3 | 4.49 | 2.26 | .62 | .21 | .56 | .29 |
| 25 | 5 | 4 | 4.81 | 2.20 | .58 | .24 | .64 | .37 |
| 26 | 6 | 3.75 | 5.38 | 2.11 | .59 | .09 | .54 | .36 |
| 27 | 5 | 4 | 4.99 | 2.21 | .50 | .15 | .54 | .33 |
| 28 | 5 | 4 | 5.15 | 2.15 | .55 | .14 | .41 | .18 |
| 29 | 7 | 2 | 6.50 | 1.99 | .45 | .45 | .73 | .42 |
| 30 | 4 | 3 | 4.54 | 2.33 | .51 | .25 | .58 | .30 |
| 31 | 4 | 4 | 4.57 | 2.39 | .46 | .34 | .68 | .39 |
| 32 | 8 | 1 | 7.39 | 1.49 | .55 | .02 | .32 | .24 |
| 33 | 3 | 3 | 3.38 | 1.86 | .55 | .02 | .08 | -.03 |
| 34 | 4 | 3 | 3.98 | 2.00 | .49 | .05 | .42 | .25 |
| 35 | 7 | 3 | 6.39 | 1.95 | .49 | .45 | .72 | .33 |
| 36 | 6 | 2 | 6.05 | 1.89 | .48 | .03 | .47 | .35 |
| 37 | 5 | 4 | 5.04 | 2.31 | .37 | .17 | .69 | .44 |
| 38 | 5 | 3 | 4.69 | 2.03 | .41 | .17 | .65 | .38 |

Me = median, IQR = interquartile range, M = mean, SD = standard deviation, RIT = Pearson correlation between item and total score (reverse items were recoded), RIR = Pearson correlation between item and rest of items (reverse items were recoded), ULI = Upper-Lower Index (reverse items were recoded), Alpha Drop = Cronbach's α of test without given item (reverse items were recoded).
